# Early lactate-guided therapy in cardiac surgery patients: a randomized controlled trial

**DOI:** 10.1186/cc13360

**Published:** 2014-03-17

**Authors:** M Sundin, J Almeida, E Osawa, F Galas, M Gaiane, S Zefferino, L Camara, LG Galas, L Hajjar

**Affiliations:** 1 Heart Institute, São Paulo, Brazil; 2 Instituto do Cancer do Estado de São Paulo, Brazil

## Introduction

It is unknown whether lactate monitoring aimed to decrease levels during the first hours in patients undergoing cardiac surgery improves outcome. The aim of this study was to evaluate the effect of lactate monitoring and resuscitation directed at decreasing lactate levels in patients admitted to the ICU in the first 8 hours with lactate level ≥3.0 mEq/l.

## Methods

Patients were randomly allocated to two groups. In the lactate group, treatment was guided by lactate levels with the objective to decrease lactate by 20% or more per 2 hours for the initial 8 hours of ICU stay. In the control group, the treatment team had no knowledge of lactate levels (except for the admission value) during this period. The primary outcome measure was the incidence of complications in 28 days.

## Results

The lactate group received more fluids and dobutamine. However, there were no significant differences in lactate levels between the groups. The rate of complications was similar between groups (11% vs. 7%, *P *= 0.087). Length of ICU stay was higher in the lactate group (3.5 vs. 2.4 days, *P *= 0.047) when compared with the control group.

## Conclusion

In patients with hyperlactatemia on ICU admission, lactate-guided therapy did not reduce complications and was related to a longer ICU length of stay. This study suggests that goal-directed therapy aiming to decrease initial lactate levels does not result in clinical benefit.

